# Finding a Better Solution for Asthma Control in Children

**DOI:** 10.7759/cureus.3344

**Published:** 2018-09-22

**Authors:** Shaheen Sombans, Petras Lohana

**Affiliations:** 1 Internal Medicine, Bharati Vidyapeeth University Medical College and Hospital, Pune, IND; 2 Internal Medicine, Liaquat University of Medical and Health Sciences Hospital, Karachi, PAK

**Keywords:** asthma, children

## Abstract

Asthma is a chronic respiratory condition that is very common among children. With proper management and follow-ups, this condition can be easily controlled in a child. However, there are several factors that are negatively influencing a proper care of asthmatic children throughout the world. In this mini-review, the roles of the parents, the schools and the child himself were analyzed and several possible solutions to improve those hurdles were discussed.

## Introduction and background

Asthma is a chronic respiratory condition with a genetic as well as environment predisposition that is characterized by chronic inflammation leading to reversible airflow obstruction and hyper-responsiveness [1-3]. The incidence of asthma in the pediatric population varies geographically and has been reported as 5.3% in India [4], 15.8% in Pakistan [5], 22.0% in the Dominican Republic [6], and 33.1% in Peru [7]. Asthma can affect the development of a child both at home and at school. It is thus vital to properly manage the condition in children. In this mini-review, we briefly evaluated possible changes by the parents, the schools, and the children, that can be brought about to tackle the different hurdles.

## Review

The role of parents

There are many hurdles for an adequate control of asthma in many underdeveloped and developing countries. The low educational background of the majority of parents in developing countries can prevent them from properly identifying the symptoms of asthma early on. It is thus vital to encourage some form of educational campaigns that are easily understood. The stigmatization of asthma in children should also be removed [8]. Parents should be able to understand that asthma is a condition that can be easily managed with correct changes in lifestyle and treatment. The fear of an early use of corticosteroids in a child by parents should be addressed by the physicians [9]. As religion is a major influencing factor in less developed countries, many religious leaders can also help in such campaigns [10].

It is estimated that the death rate from asthma among children is 0-0.7 per 100,000 [11]. Since the quality of air and air pollution can predispose to asthma, children in big cities should be encouraged to take preventive measures early on. A study done in Chongqing, China showed that even short-term exposure to PM10 (particulate matter 10 micrometers or less in diameter) or PM2.5 (particulate matter 2.5 micrometers or less in diameter), as well as sodium dioxide, nitrogen, and carbon monoxide, could provoke hospital visits for asthma in children [12]. Children in big cities with a high level of pollution should be monitored for signs of asthma. A study by Kainuma et al. even found that the use of filtering masks during sleep can help reduce the exposure of allergens and decrease the risk of asthma attacks in children [13]. Smoking has also been linked with asthma in children. Exposure to second-hand smoking should also be avoided and smoking parents should be advised to wash their hands and change their clothes before interacting with a child [14].

The role of schools

The lack of good medical care in many underdeveloped and under-served areas of the world can lead to a poor compliance as well. One possible solution to this issue would be to encourage the ministry of health and the ministry of education in such countries to conduct regular biannual visits of physicians in schools to correctly screen for children with asthma and monitor their compliance and response to therapy [15]. Rapid and correct management is vital for cases of acute exacerbation of asthma. School teachers should also be well trained to identify any symptoms of asthma in a child. Any wheezing and shortness of breath should raise concern in a child at school and immediate help should be sought. Children suffering from uncontrolled asthma have a disadvantage at school [16]. Having school nurses who can help the child and set up a good care plan is vital. However, it has been reported that in the United States only 36% of schools have a full-time nurse [17]. The schools should also monitor the rate of absenteeism in asthmatics and work with the parents for a better solution to help the child.

The child himself

Children are often reluctant to admit that they have asthma. Teenagers have often been reported to stop taking their medications as they are embarrassed [18]. This is more common in males than in females [19]. Such behavior can lead to non-compliance with the therapy that can lead to poor outcomes. A study conducted by Walker and Reznik in 2014 showed that minority children in the Bronx reported: "inappropriate in-school management of asthma symptoms, poor asthma control, lack of accessible medication, and stigma around publicly using asthma medication" [20]. The schools, as well as pediatricians, should strongly raise such questions with the child and proper encouragement for the use of the drugs provided. They should be correctly informed about the risks of stopping or skipping their medications and should be encouraged to meet other children who are also in a similar situation. Group meetings between asthmatics and school counselors can also help understand any difficulties they might be experiencing in school and how they can be overcome [21-23].

A child with asthma and currently under treatment should be compliant with his medications. A child who has been given a rescue inhaler should always bring it with him, even at school. The school can help parents with the follow-up and compliance by regularly investigating the medications the child is taking. The child should also be adequately educated about the uses of the medications. He should learn the doses he is taking and if any dose is missed, he will be able to inform the parents.

## Conclusions

Asthma is currently one of the most common respiratory conditions in children. While there are multiple challenges in underdeveloped and developing countries in terms of proper care of pediatric asthma, the hurdles can be overcome if parents, health care providers, teachers as well as governmental and non-governmental organizations work together.
